# Regulatory interdependence of myeloid transcription factors revealed by Matrix RNAi analysis

**DOI:** 10.1186/gb-2009-10-11-r121

**Published:** 2009-11-02

**Authors:** Yasuhiro Tomaru, Christophe Simon, Alistair RR Forrest, Hisashi Miura, Atsutaka Kubosaki, Yoshihide Hayashizaki, Masanori Suzuki

**Affiliations:** 1 RIKEN Omics Science Center, RIKEN Yokohama Institute 1-7-22 Suehiro-cho, Tsurumi-ku, Yokohama, Kanagawa 230-0045, Japan; 2 International Graduate School of Arts and Sciences, Yokohama City University, 1-7-29 Suehiro-Cho, Tsurumi-Ku, Yokohama 230-0045, Japan; 3 The Eskitis Institute for Cell and Molecular Therapies, Griffith University, Brisbane Innovation Park, Don Young Road, Nathan, QLD 4111, Australia

## Abstract

**Background:**

With the move towards systems biology, we need sensitive and reliable ways to determine the relationships between transcription factors and their target genes. In this paper we analyze the regulatory relationships between 78 myeloid transcription factors and their coding genes by using the matrix RNAi system in which a set of transcription factor genes are individually knocked down and the resultant expression perturbation is quantified.

**Results:**

Using small interfering RNAs we knocked down the 78 transcription factor genes in monocytic THP-1 cells and monitored the perturbation of the expression of the same 78 transcription factors and 13 other transcription factor genes as well as 5 non-transcription factor genes by quantitative real-time RT-PCR, thereby building a 78 × 96 matrix of perturbation and measurement. This approach identified 876 cases where knockdown of one transcription factor significantly affected the expression of another (from a potential 7,488 combinations). Our study also revealed cell-type-specific transcriptional regulatory networks in two different cell types.

**Conclusions:**

By considering whether the targets of a given transcription factor are naturally up- or downregulated during phorbol 12-myristate 13-acetate-induced differentiation, we could classify these edges as pro-differentiative (229), anti-differentiative (76) or neither (571) using expression profiling data obtained in the FANTOM4 study. This classification analysis suggested that several factors could be involved in monocytic differentiation, while others such as MYB and the leukemogenic fusion MLL-MLLT3 could help to maintain the initial undifferentiated state by repressing the expression of pro-differentiative factors or maintaining expression of anti-differentiative factors.

## Abstract 2

The knockdown of 78 transcription factors in differentiating human THP-1 cells using matrix RNAi reveals their interdependence

## Background

The importance of transcription factors (TFs) in regulating gene expression and determining cellular states is well known. However, in mammalian systems, the lists of target genes and the interdependence of most TFs are still poorly defined. Due to the connectivity of transcriptional regulatory networks (TRNs), the activities of any given TF can be regulated by many other factors. Approaches to identify TF-target gene dependencies include TF binding site (TFBS) predictions (for the 200 or so factors with well defined motifs) [1], whole-genome chromatin immunoprecipitation (ChIP) experiments [2,3] and perturbation experiments that use overexpression [4], knockdown [5-7] or knockout [8,9] of a TF in question followed by expression profiling to determine TF-target gene dependencies.

Considering the short nature of most TFBSs, their prediction is noisy, with large numbers of false positives. Most researchers focus on experimentally identified promoter regions rather than the whole genome and use inter-species conservation information to improve the signal-to-noise ratio of TFBS predictions [10]. Another issue that affects the quality of these predictions is that different TFBS position weight matrices have different predictive power. This can be due to the variable length of TFBSs, the variability in information content along the length of the motif and the varying quality (and depth of known true positives) of the data used to generate the matrices in the first place.

ChIP analysis, in particular ChIP-seq [2,11], is becoming a gold standard for determining TF-target gene associations; however, these are currently limited by the number of ChIP-quality antibodies available. In addition, it is not entirely clear if the binding of a factor to a genomic region is sufficient to infer that the factor regulates genes within that region [12,13]. On the other hand, perturbation experiments using RNA interference (RNAi) knockdown can be virtually applied to any TF because specific small interfering RNAs (siRNAs) should be available to silence the target TF genes. The effects of decreasing the concentration of a given TF on the expression of other genes can be directly measured; if an effect is observed, the factor either directly or indirectly regulates expression of that particular target gene [7].

We have developed a systematic RNAi-perturbation analysis system, named Matrix RNAi, in which siRNA knockdown and quantitative real-time RT-PCR (qRT-PCR) are used in combination on the same set of TFs to determine their interdependencies [7]. The Matrix RNAi approach has already been proven to be very useful to identify TF-TF gene regulatory relationships of a small set of regulators and the regulatory regions of some of their target genes that are involved in combinatorial transcriptional regulation [14]. This prompted us to assess in more detail the general applicability of the Matrix RNAi experimental system to inter-TF TRN analysis, including combinatorial regulation. Here we exploit the Matrix RNAi analysis system to examine the interdependency of 78 TFs in the human acute myeloid leukemia (AML) cell line THP-1 [15]. THP-1 cells can be induced to differentiate into macrophage-like cells by activation with phorbol 12-myristate 13-acetate (PMA) or vitamin D3 and are widely used as the experimental model of monocytic differentiation [16]. The dynamic transcriptional network of THP-1 cells during PMA-induced differentiation was recently analyzed intensively by the FANTOM4 consortium [17]. The majority of factors targeted in the present study are known or suspected to be TFs with roles in myeloid cell lineages and several are implicated in leukemia. In addition, we include factors predicted by the FANTOM4 consortium as important in regulating monocytic differentiation [17]. Although these TF genes have been implicated in myeloid cell functioning, how and whether they work together has not been previously addressed. In the present Matrix RNAi approach, we knocked down these 78 TFs and then measured the perturbation effects on a panel of 96 genes (91 TF and 5 non-TF genes), including the original 78, by qRT-PCR.

This approach extracted 876 significant TF-TF gene candidate edges from a total of 7,488 possible combinations. Out of these, 654 were activating edges (that is, knockdown of one TF led to a decrease of expression of another) and 222 were repressing edges (that is, knockdown of one TF led to an increase of expression of another). Using TFBS predictions in the proximal regulatory regions and targeted ChIP, we then attempted to identify which of these edges were most likely to be direct ones. This approach is successfully applied to build the framework of an inter-TF TRN in the monocytic leukemia cell and we discuss its structure in relation to leukemia, myeloid differentiation and maintenance of the undifferentiated state.

## Results and discussion

### Target gene selection and optimization of siRNA transfections

We carried out RNAi knockdown of individual TFs that were predicted to play a role in PMA-mediated differentiation of THP-1 cells to assess the impact on genome-wide gene expression using Illumina whole genome microarrays. This was used to experimentally confirm the gene dependency predicted using the expression-weighted TFBS predictions for the TFs targeted (see [17] for analysis of the transcriptional network of THP-1 cells during PMA-induced differentiation). It also allowed us to explore the role of each factor in the differentiation process, including those lacking currently available information about their TFBS motifs. In the present study, we focused on TF-TF gene dependencies (edges) and for this purpose used qRT-PCR measurements, enabling us to more precisely measure the responses or perturbations of a panel of TF genes to the knockdown of individual TFs. The TFs targeted include many of those previously reported to be involved in differentiation and myeloid leukemia and others predicted to play a role in differentiation from our TFBS analyses [17]. We also supplemented this list with a set of nine siRNAs against TFs commonly expressed in THP-1 cells and HepG2, a human hepatoma cell line, to explore network variation in different cellular systems (Additional data file 1).

To begin the Matrix RNAi experiments, we assessed the knockdown efficiency and reproducibility of a panel of 91 siRNAs targeting 86 TF and 5 non-TF genes. Chemically modified 'stealth siRNAs' (Invitrogen, Carlsbad, CA, USA) that were designed to reduce siRNA-induced interferon responses and off-target effects were used in these experiments and several were tested for each TF to select individual siRNAs causing at least 60% downregulation (as assessed by qRT-PCR). For 83 TF genes we were able to find an siRNA that achieved an average knockdown of more than 60%. We further filtered these siRNAs according to two criteria - standard deviation (SD) and *P*-value of the expression change (ΔΔC_T_; see 'Expression analysis' in Materials and methods) - for each TF gene silenced: significant TF knockdown requires the average ΔΔC_T _to be greater than 2 SDs, with a *P*-value of less than 0.05. These requirements excluded another five TFs and we continued our analysis with the remaining 78 TFs (Additional data file 1). We have also confirmed that the knockdown of a random selection of 14 of these TF genes by specific siRNAs resulted in significant reductions in the levels of the corresponding TF proteins (Additional data file 2).

### Matrix RNAi analysis

Using the 78 TF-specific siRNAs described above, we then carried out a 78 × 96 matrix RNAi-perturbation analysis, where the expression of 91 TF and 5 non-TF genes were assessed by qRT-PCR. Each transfection was carried out in quadruplicate and RNAs were harvested 48 hours after transfection. RNAi knockdown efficiency and expression perturbation of each TF gene were evaluated in a similar way to the determination of the siRNA activities. The siRNA must produce at least 60% perturbation, and for an edge to be identified as significantly perturbed, the average expression change (ΔΔC_T_) of each of the target TF genes needs to be greater than 2 SDs, with a *P*-value of less than 0.05 (Additional data file 3).

From a total of 7,488 possible edges from the 78 × 96 matrix (Additional data file 4), we identified 876 putative edges that were significantly perturbed in our analysis (Additional data files 5 and 6 and Figure S1 in Additional data file 7). Out of these, 654 were activating edges (that is, knockdown of one factor led to a significant decrease in the expression of another) and 222 were repressing edges (TF knockdown led to an increase in the expression of another). The ratio of activating to repressing edges is similar to what we have observed previously in the Matrix RNAi analysis of TFs enriched in HepG2 cells [7]. In THP-1 cells, the majority of knockdowns led to downregulation of target genes, indicating that these TFs work as activators. On the other hand, knockdown of a number of other TF genes, for example, *MYB *and *NFE2L1*, led to upregulation of multiple genes. Such perturbations biased in favor of upregulation suggest that these factors may work primarily as repressors in THP-1 cells: *MYB*-specific siRNA downregulated and upregulated 5 and 15 genes, respectively, while *NFE2L1 *knockdown downregulated and upregulated 4 and 16 genes, respectively.

The TF set examined in the present Matrix RNAi analysis contains a number of redundant (paralogous) TFs - for example, PPARD and PPARG, STAT1 and STAT3, RARA and RARG, RXRA and RXRB - that could back up the other family member knocked down, as observed in TF knockout budding yeast [18]. If this is also the case in the RNAi knockdown and mammalian transcriptional regulatory system, knockdown of a TF could be compensated by its paralogous TF(s), resulting in no or little perturbation of expression of its target genes, which would lead to underestimation of the perturbation results. However, we found several examples that knockdown of each of the redundant TFs leads to expression perturbations of their common genes. For example, knockdown of PPARD and PPARG, which are known to target the same recognition sequence (a PPAR response element), were found to share common target genes to be perturbed. All of the different STATs bind to regulatory elements with the common core motif [19] and our Matrix RNAi analysis includes two STAT family members, STAT1 and STAT3. Their knockdown upregulated five common genes but also downregulated one gene (*EGR1*). Knockdown of RXRA and RXRB, which also share common sequence motifs, resulted in perturbation of four common genes in the same direction. Moreover, in the knockdown of these pairs of redundant TFs, no reciprocal rescue of the paralogous counterparts was observed in our Matrix RNAi study. These findings suggest that backup by redundant paralogous TFs may not be a prevailing mechanism in the Matrix RNAi system studied here. In the study on budding yeast [18], backup by paralogous genes was mostly examined in yeast cells in which the target genes had been knocked out and functional compensation by their counterpart paralogs had already been established. On the other hand, the knockdown examined in the present study results in abrupt downregulation of each TF gene for which backup by a paralogous counterpart may not have already been established. Perturbation data from non-TFs may help to distinguish the effects of transcriptional and non-transcriptional regulatory mechanisms on the changes in gene expressions upon knockdown. Our Matrix RNAi assays included five non-TF members (BCL2, CR595360, FUS, NRAS, and PRPF31). RNAi knockdown of BCL2 led to a slight change in the expression level of only a single gene (*SPI1*) out of 78 TF genes (except self knockdown). In contrast, knockdown of the other non-TF genes, CR595360, *FUS*, *NRAS*, and *PRPF31*, caused changes in the expression of varying numbers of TF genes (7, 16, 12 and 12, respectively). These are clearly non-transcriptional and/or indirect transcriptional regulatory effects.

In a similar fashion, these non-TF genes were also differentially affected by knockdown of TFs: CR595360, *BCL2 *and *NRAS *genes were significantly perturbed by 21, 13 and 12 TFs, respectively. In contrast, knockdown of only a single TF affected the expression of the *FUS *and *PRPF31 *genes. These non-TF genes were perturbed by 9.6 TFs on average. On the other hand, 78 TF genes were significantly perturbed by knockdown of 10.6 TFs (except self repression) on average. This very small difference in the average number of TFs that caused significant perturbations in the expression of TF and non-TF genes is indicative of a small bias of perturbation by TF knockdown toward TF genes.

It is interesting to note that CR595360 encodes an antisense RNA against the *GATA2 *gene [20] but its function is unclear. We found that GATA2 knockdown caused a significant downregulation of CR595360 expression, raising the possibility that the TF may stimulate antisense RNA expression in growing THP-1 cells. In contrast, the regulation of *GATA2 *expression by CR595360 was not observed.

### Characteristics of the perturbation network composed of 876 TF-TF gene edges

From our perturbation analysis data identifying a total of 876 significant perturbation edges, the expression of 92 genes were affected by knockdown of another factor, while the expression of *CTCF*, *HNF4G*, *MAZ *and *NFE2L1 *was not affected by any other TF gene knockdown. Knockdowns of 77 TF genes affected the expression of at least one other TF gene, while knockdown of the gene *SREBF1 *did not affect the expression of any other TF gene in the matrix.

**Figure 1:**
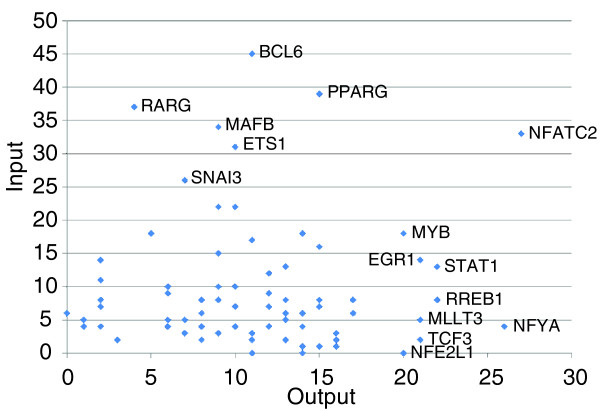
Dot-plot showing the relationship between the number of input connections and output connections. The number of input and output connections corresponds to that of TFs regulating a given TF gene and that of TF genes regulated by a given TF, respectively. Only significant edges in the perturbation network identified by the Matrix RNAi analysis are counted.

We would like to intend: Comparing the number of genes affected by the knockdown of a given TF with the number of other TFs whose knockdown affected the expression of the given TF gene, we detected highly multiple input and output nodes (Figure 1). The expression of each of the genes *SNAI3*, *RARG*, *ETS1*, *MAFB*, *PPARG*, *BCL6 *and *NFATC2 *was affected by knockdown of any TF of a set of more than 25 (the sets of TFs used for each gene did not include the TF encoded by that gene itself). Conversely, knockdown of *MYB*, *MLLT3*, *EGR1 TCF3*, *RREB1*, *STAT1*, *NFYA *and *NEF2L1 *affected the expression of more than 20 other TF genes. Notably, *NFATC2 *was both highly 'in-connected' and 'out-connected', suggesting that it plays a role as a giant hub.

These numbers may be considered to be too high to explain the direct transcriptional regulation by these TFs. Because the process of enrichment or mining of potential candidates for transcriptional regulatory edges intrinsically requires the setting of appropriate thresholds for that purpose, the occurrence of false positives as well as false negatives may be inevitable; thus, the high number of positive edges may include a considerable number of false positives.

### Pro-differentiative and anti-differentiative edges in the network

**Figure 2:**
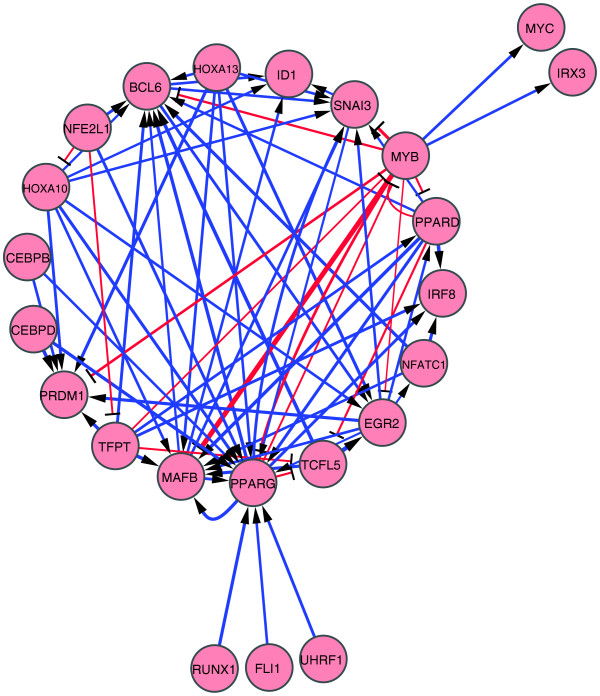
Perturbation networks of both pro-differentiative and anti-differentiative TF genes were selected as nodes for this network. To depict the putative network, only significant edges (mean of ΔΔC_T _> 2 SD and *P *< 0.05) were extracted based on Matrix RNAi data (Additional data file 5). The networks were drawn by Cytoscape [41]. In these networks, TFs and TF genes regulated by them are not distinguished from each other, but the nodes emitting and accepting an arrow represent putative regulators and regulated genes, respectively. Arrowheads and blue lines indicate stimulatory regulation. T-shaped heads and red lines indicate repressive regulation. Line widths indicate perturbation magnitude.

To further explore the network, we broke it down into two sub-networks composed of pro-differentiative and anti-differentiative edges using the FANTOM 4 data set. In the FANTOM 4 data set, 64 and 34 TFs were most highly expressed in the undifferentiated and differentiated states, respectively, and thus categorized as anti-differentiative and pro-differentiative [17]. Of our 96 target genes, 11 were classified as a pro-differentiative TF gene and 13 as an anti-differentiative TF gene (Additional data file 1). On the other hand, our matrix RNAi analysis can discriminate activation from repression among regulatory edges to target TF genes; we thus classified the regulatory edges as pro-differentiative and anti-differentiative. A pro-differentiative edge represents activation of a pro-differentiative TF gene or repression of an anti-differentiative TF gene. By contrast, an anti-differentiative edge represents activation of an anti-differentiative TF gene or repression of a pro-differentiative TF gene. Figure 2 summarizes the perturbation network composed of both pro-differentiative and anti-differentiative edges.

**Figure 3:**
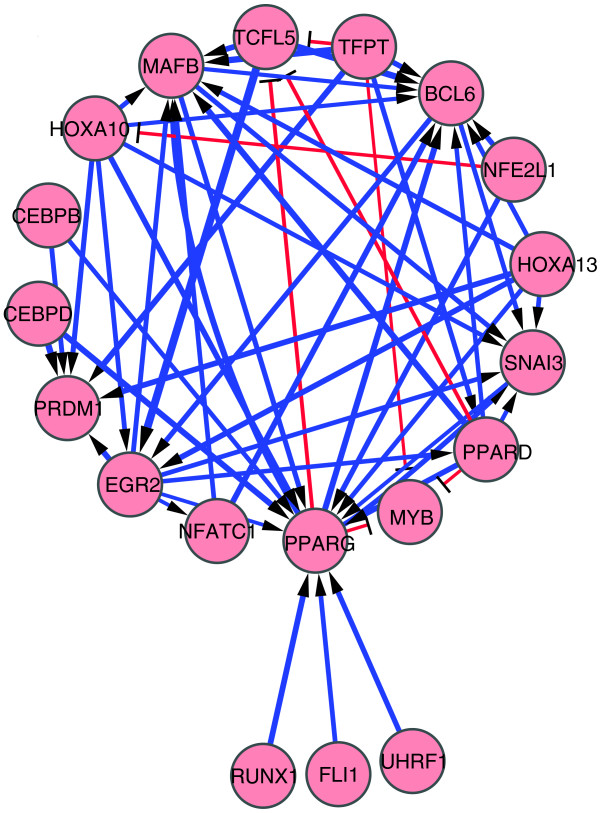
Perturbation network of pro-differentiative edges. Only pro-differentiative TF genes were selected as nodes for this network. See the legend of Figure 2 for extraction of regulatory edges and network drawing.

Using the above definitions, we identified 229 pro-differentiative edges from a total of 876 significant perturbation edges revealed by knockdown of 65 factors and drew a perturbation network composed of these pro-differentiative edges (see Figure S2 in Additional data file 7 for all the pro-differentiative edges and Figure 3 for those identified among the 11 pro-differentiative and the 13 anti-differentiative TFs and/or TF genes). In this network, most edges detected were activating: for example, *BCL6*, *PRDM1*, *PPARG *and *MAFB *levels were all significantly reduced upon knockdown of multiple factors and are normally upregulated during monocytic differentiation of THP-1 cells. However, some factors such as MYB and TCFL5, which are both downregulated during differentiation, appeared to be repressed by multiple factors. Of note, HOXA13, CEBPB and CEBPD are restricted to the pro-differentiative edge network, strongly suggesting their positive regulatory roles in THP-1 cellular differentiation.

**Figure 4:**
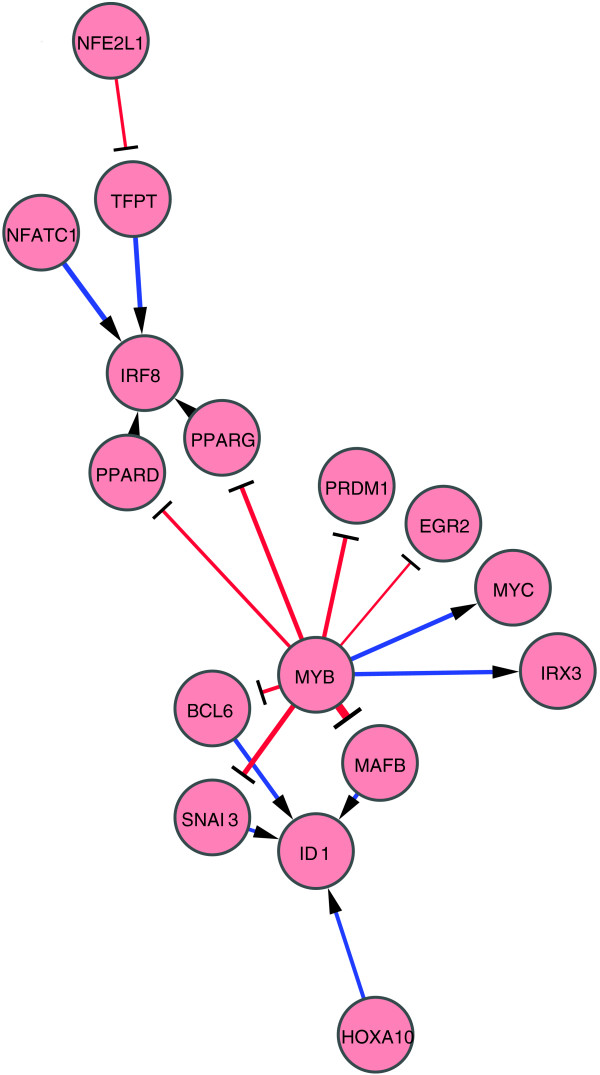
Perturbation network of anti-differentiative edges. Only anti-differentiative TF genes were selected as nodes for this network. See the legend of Figure 2 for extraction of regulatory edges and network drawing.

We also drew a network composed of 76 anti-differentiative edges generated by the knockdown of 44 factors (see Figure S3 in Additional data file 7 for all the anti-differentiative edges and Figure 4 for those identified among the 11 pro-differentiative and the 13 anti-differentiative TFs and/or TF genes). In this network, MYB appears to play in a central position in repressing expression of *BCL6*, *EGR2*, *SNAI3*, *PPARD*, *PPARG*, *PRDM1 *and *MAFB*, all of which are normally upregulated during PMA-induced differentiation. Of note, *ID1 *and *IRF8*, which are known as negative transcriptional regulators [21,22], are likely positively regulated by several TFs in the anti-differentiative edge network, suggesting their involvement in the maintenance of the undifferentiated (anti-differentiative) state of monocytic cells.

### MYB and MLLT3 synergistically function as anti-differentiative factors in THP-1 cells

An siRNA targeting the leukemogenic fusion of MLL-MLLT3, found in THP-1 cells [23], upregulated expression of *MAFB *and downregulated that of both *HOXA10 *and *HOXA13 *(Figure S3 in Additional data file 7). Positive regulation of several HOX genes (*HOXA3*, *HOXA7 *and *HOXA10*) by MLL-MLLT3 has been previously demonstrated using antisense oligonucleotides [24]. *HOXA10 *overexpression results in AML and prevents lymphomyelopoiesis [25,26] and *HOXA13 *is the known target of another leukemogenic fusion related to AML [27]. Our finding indicates that MLL-MLLT3 plays a role in extensive positive regulation of *HOXA *genes highly connected to AML pathology.

The repression of the gene *MAFB *by both MYB and MLL-MLLT3 is also of interest. *MAFB *overexpression induces monocytic differentiation [28], and there is an antagonistic relationship between MYB and MAFB, such that MYB is thought to maintain the undifferentiated state by direct binding to sumoylated MAFB [29]. In our experiments, MYB also affected the transcript level of *MAFB*, but to our knowledge, this is the first report of a role for MLL-MLLT3 in the regulation of *MAFB *expression level. Interestingly, MYB and MLLT3 regulated both 'increased' and 'decreased' genes in PMA-induced differentiation, but every edge was anti-differentiative (Figure S3 in Additional data file 7). When MYB was knocked down, THP-1 cells initiated similar changes to those observed during PMA-induced differentiation, such as cell adhesion to the culture dish. Although weaker than with MYB knockdown, this change in the adherence property of THP-1 cells was also found with the knockdown of MLLT3, which is constantly expressed during PMA-induced differentiation. Based on the similarity of the regulatory and physiological outcomes of knocking down MYB and MLLT3, we assumed that these two factors might function synergistically. To explore this possible synergistic effect, we performed a double knockdown of MYB and MLLT3 in THP-1 cells. The effect of the double knockdown on the cell adhesion ability was slightly enhanced compared with that of single knockdown by specific siRNA targeting of either MYB or MLLT3 (Figure S1 in Additional data file 8). Next, we searched for TF genes commonly regulated by these two TFs and found that four genes, including *IRX3 *and MAFB, were double-regulated by them (Figure S2 in Additional data file 8). *ETS1 *and *RARG *were also regulated by both of these TFs but in opposite directions, consistent with the result of the perturbation analysis showing that MYB and MLLT3 work primarily as a repressor and a transcriptional stimulator, respectively, in THP-1 cells (Figure S3 in Additional data file 7). Although the functions of IRX3 and RARG are unknown in myeloid cells, these TFs are known to be involved in differentiation processes in neurons [30] or in responses to retinoic acid, a potent differentiation inducer in THP-1 cells [31]. Moreover, ETS1 is thought to play significant roles in hematopoiesis [32]. In addition to the important function of MAFB, synergistic regulation of these three TF genes by MYB and MLLT3 suggests their possible involvement in the regulation of THP-1 cellular differentiation.

### Identification of direct regulatory edges by X-ChIP/qRT-PCR

To predict putative direct regulatory edges, we extracted the tentative FANTOM4 TFBSs in the promoter regions of all 96 TF genes. Motifs were available for 28 out of 83 factors and a total of 83 edges, which could potentially be directly regulated by these factors. We also checked the sequences -5,000 bp to +1,000 bp from the transcription start sites (TSSs) of all 96 TF genes with a TFBS data set from the TRANSFAC database. As a result, TFBS motifs of 35 TFs were found and detected in 233 of the 417 edges representing the upstream regions of the target genes.

**Figure 5:**
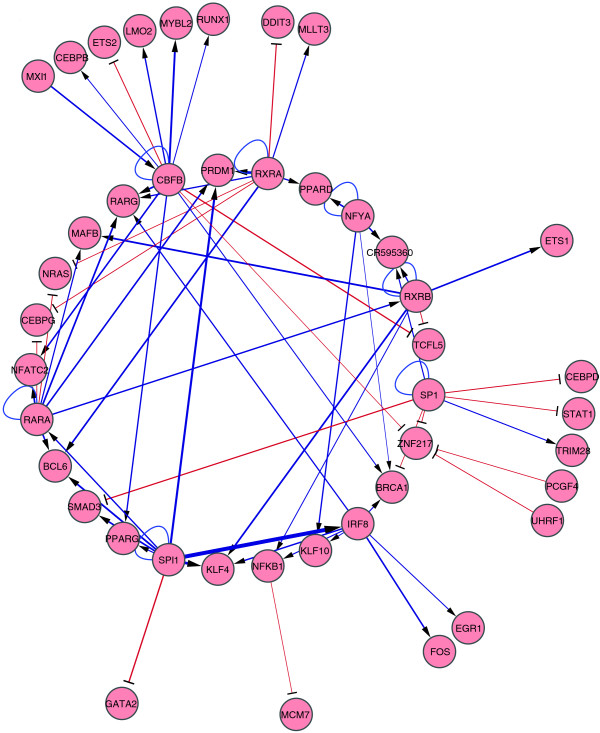
ChIP-validated network. The genes predicted to be regulated by any of 12 TFs (CBFB, IRF8, NFKB, NYFA, MXI1, PCGF4, RARA, RXRA, SP1, SPI1, RXRB and UHRF1) were examined for TF binding to their proximal promoter regions by X-ChIP/qPCR. Seventy out of 113 edges were validated as TF-binding positives, strong candidates for direct regulatory edges.

To experimentally identify direct regulatory edges in the TRN predicted by the Matrix RNAi analysis, we carried out cross-linking chromatin immunoprecipitation (X-ChIP)/qRT-PCR analysis for 12 TFs: CBFB, NFKB, NFYA, MXI1, PCGF4, RARA, RXRA, RXRB, SP1, SPI1, IRF8, and UHRF1. The Matrix RNAi analysis identified a total of 113 target genes for these 12 factors as components of significant edges, and potential TFBSs were predicted in the proximal promoter regions of all of these target genes. ChIP/qRT-PCR analysis confirmed that 70 of these target genes were bound by a corresponding factor within 500 bp of their TSS. These direct regulatory edges supported by perturbation and ChIP experiments are summarized in Figure 5 (see Additional data file 9 for the positive TF binding data).

We performed false discovery rate (FDR) analysis and calculated q-values of gene perturbations. Without considering the SD values, we detected 1,684 edges with a *P*-value < 0.05 and 1,122 edges with a q-value less than 0.05 (not including edges for auto-perturbation; Figure S1 in Additional data file 10). We found 876 significant perturbation edges with a *P*-value < 0.05 and a mean > 2 × SD, and after FDR analysis 579 edges with a q-value < 0.05 remained, indicating that the FDR analysis filtered out approximately 33.9% of the perturbation edges (Figure S1 in Additional data file 10). This may reflect the inclusion of a significant number of false positives in the whole perturbation analysis.

Next, the validity of the filtering of significant edges using q-values in reference to TF-binding data was assessed. We calculated the ratio of TF binding-positive to TF binding-negative edges with a *P*-value < 0.05 and a mean > 2 × SD and the ratio for those with a *P*-value < 0.05, a q-value < 0.05 and a mean > 2 × SD; these were 0.619 and 0.588, respectively (Figures S2 and S3 in Additional data file 10). This difference in the percentage of binding-positive edges by adoption of a q-value threshold (< 0.05) may be less significant in terms of statistics. The number of binding-positive edges was decreased to 57.1% (from 70 to 40 edges) by adoption of a q-value threshold and the decrease (42.9%) is significantly larger than the decrease (33.9%; from 876 to 579) in the number of edges with a *P*-value < 0.05 and a mean > 2 × SD after the adoption of a q-value threshold (< 0.05). Moreover, there is no significant difference in the distribution of q-values for gene perturbation between the binding-positive and binding-negative edges (Figure S4 in Additional data file 10). Taken together, filtering by the FDR threshold failed to result in enrichment of perturbation- and binding-positive edges and appears to generate many false negatives.

We have previously shown that validation of binding of TFs to the proximal promoter regions of their potential gene targets leads to successful reduction of the ratio of false positives to false negatives [7]. In the Matrix RNAi assays of the relationships between TFs in HepG2 cells, binding-positive edges constitute 75% of the perturbation-positive edges, suggesting that the filtering by TRF binding data obtained in ChIP assay may enrich the significant edges. Based on the assumption that edges that are both perturbation- and binding-positive may be much more valid compared with the edges inferred by only either of perturbation or binding assay, 70 perturbation and binding positive edges appear to be highly reliable edges (Figures S2 in Additional data file 10).

*BCL6 *was not significantly perturbed by 7 out of 12 TFs that were used for the X-ChIP analysis and was slightly affected by the knockdown of PCGF4. On the other hand, three (SPI1, RARA and RXRA) out of the four TFs whose knockdown greatly affected (downregulated) *BCL6 *gene expression were demonstrated to bind the upstream region of *BCL6 *(Additional data file 9, X-ChIP/qPCR assay data). The considerable overlap between *BCL6 *gene expression perturbations caused by TFs and TF binding to the *BCL6 *proximal promoter region strongly suggests that *BCL6 *is actually regulated by multiple TFs in growing THP-1 cells. Moreover, the genes *PPARG*, *RARG*, *PRDM1 *and *MAFB *were bound and perturbed by 2, 4, 3 and 2 out of 12 TFs tested by ChIP analysis, respectively, but were perturbed by only 2, 0, 1 and 0 TFs other than these binding- and perturbation-positive TFs (Additional data file 9). The observed high concordance between TF-binding and perturbation strongly suggests direct regulation of these genes. Many TFs can cooperate to regulate transcription of a mammalian gene by a combinatorial regulation mechanism. Indeed, transcriptional regulation occurs through interaction between multiple distantly located regulatory sites that are bound by several different TFs [14,33,34], as represented by enhanceosomes, in mammalian systems. Although the number of edges evaluated by ChIP analysis in the present study is rather small, the experimental results obtained suggest that the perturbation data could be used to mine efficiently the potential candidates for functional edges in combinatorial transcriptional regulation.

### Cell type-specific network

**Figure 6:**
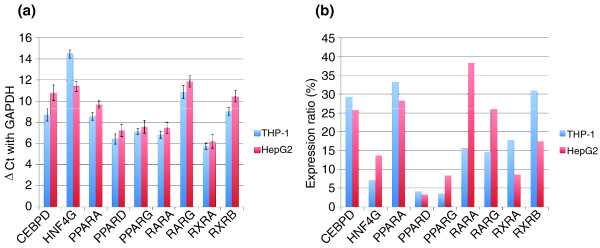
Comparison of expression levels and knockdown efficiencies for selected TFs common to THP-1 and HepG2 cells. In our previous study, human hepatoma cell line HepG2 was used to construct a Matrix RNAi experimental system for TRN analysis [7]. The Matrix RNAi data for nine TF genes (*HNF4G*, *CEBPD*, *PPARA*, *PPARD*, *PPARG*, *RARA*, *RARG*, *RXRA *and *RXRB*) was used to depict the two types of perturbation networks. **(a) **Gene expression levels in HepG2 (blue) and THP-1 (red) cells treated with negative control siRNA. **(b) **Gene expression levels in HepG2 (blue) and THP-1 (red) cells after RNAi knockdown.

**Figure 7:**
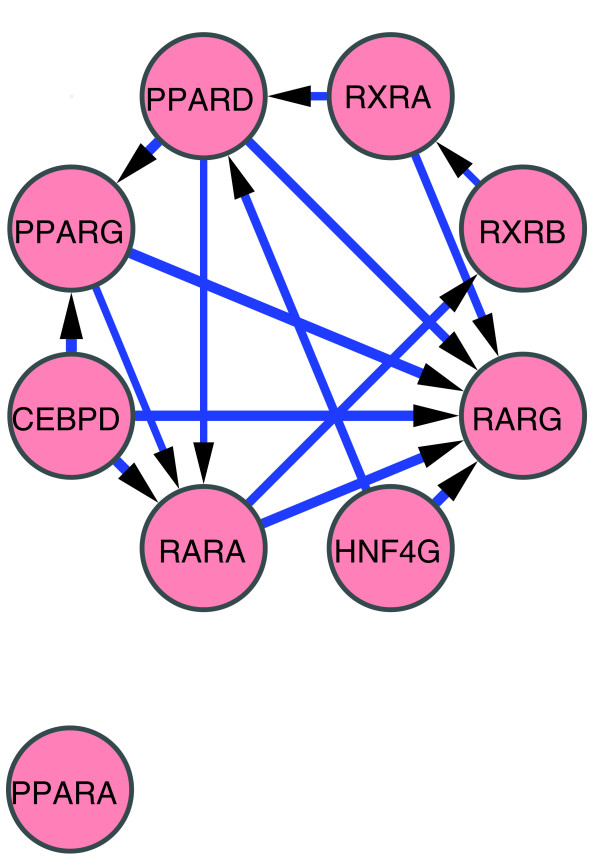
THP-1 network. Perturbation networks were constructed with only significant edges (mean of ΔΔC_T _> 2 SD and *P *< 0.05). All of the regulatory edges in this network are stimulatory.

**Figure 8:**
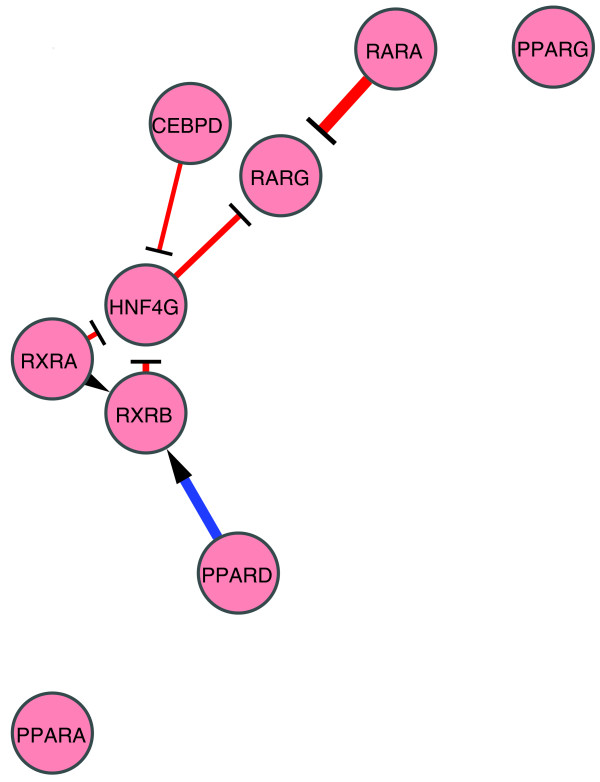
HepG2 network. Perturbation networks were constructed with only significant edges (mean of ΔΔC_T _> 2 SD and *P *< 0.05). The regulatory edge with an arrowhead and blue line indicate a stimulatory edge. T-shaped heads and red lines indicate repressive regulation. Line widths indicate perturbation magnitude.

Previously, we used Matrix RNAi with 19 TFs and 21 TF genes to study their inter-TF TRN in HepG2 cells [5]. Ten of these TFs (HNF4G, CEBPA, CEBPD, PPARA, PPARD, PPARG, RARA, RARG, RXRA and RXRB) were found to be also fully expressed in THP-1 cells. To probe the differences in TRN structure between the different cell type lineages, we compared the regulatory edges observed in HepG2 cells with those in THP-1 cells for the same set of TFs. CEBPA was excluded from this analysis as we observed poor knockdown of it in THP-1 cells. The relative expression levels of each of the remaining nine TFs were similar between THP-1 and HepG2 cells (Figure 6). Surprisingly, however, none of the edges (TF-TF gene dependencies) were the same in both cell lines (Figures 7 and 8). For example, *HNF4G *is repressed by RXRB, CEBPD and RXRA in HepG2 cells, but we observed no such dependence in THP-1 cells. On the other hand, *RARA *is activated by CEBPD, PPARD and PPARG in THP-1 cells, but we observed no such dependence in HepG2 cells. In addition, the difference in the regulatory relationship does not appear to be a function of the thresholds used for significance as less stringent thresholds also did not reveal common edges (data not shown). This suggests that the perturbation networks revealed by systematic RNAi analysis are strongly cell type dependent. It is intriguing to note that the stimulatory edges are predominant in the perturbation network of THP-1 cells and, in contrast, the repressive edges predominate in HepG2 cells, suggesting great differences in the functional roles of these TFs between these two different types of cell. Moreover, almost all of the TFs selected form regulatory circuitry and most of the regulatory edges containing retinoic acid and retinoid receptors (RARs and RXRs) are pro-differentiative in THP-1 cells, consistent with the fact that THP-1 cells differentiate into macrophages in response to all-trans retinoic acid treatment [35,36].

## Conclusions

Here, we present the first application of the large-scale Matrix RNAi approach to TRN analysis in human cells. This analysis provided a TRN of monocytic THP-1 cells growing in undifferentiated conditions as a snapshot network. These data could also be useful to find combinatorial regulatory regions that are not only proximal but also distal to the TSSs as well as the inter-TF TRN edges [14]. This network not only keeps the balance and maintains stable undifferentiated conditions, but also has the potential to initiate differentiation in response to a stimulus such as PMA. Together with the data of time-dependent changes in expression patterns of TF genes during PMA-induced differentiation derived from the FANTOM4 analysis, we have used a new approach to extract pro-differentiative and anti-differentiative edges from the significant perturbation network in static THP-1 cells that were revealed by Matrix RNAi. As a result, we successfully extracted 229 pro-differentiative and 76 anti-differentiative edges. In the anti-differentiative network, MYB functions as a key negative regulator, and in the pro-differentiative network, MAFB, PPARG and BCL6 work as large hub nodes.

In this study, we identified 876 significant edges from 7,488 possible combinations in the 78 × 96 matrix, enabling us to draw a significant perturbation network. Out of these significant edges, 654 are activating edges and 222 repressing. The average number of output and input edges for one TF were 11.3 and 9.5, respectively. In the FANTOM 4 data set, potential TFBSs were predicted around -300 bp from the TSSs in the proximal regulatory regions of the target TF genes. Eighty-three out of 394 edges were regulated by 28 TFs whose DNA-binding motifs were available. Additionally, we predicted with the Transfac database and the Match program that some TFBSs could be located in a region spanning -5,000 to + 500 bp from the TSS of each of the target TF genes. More than half (233 edges) of the 417 edges regulated by 35 TFs were supported by this analysis. Finally, we validated 70 out of 113 significant edges by X-ChIP/qPCR with specific antibodies against 12 selected TFs.

A comparison of the perturbation networks of nine TFs common to the hepatoma (HepG2) and monocytic (THP-1) cell lines showed that these were surprisingly quite different from each other, even though the expression levels of these TFs were almost similar in these two types of cell. We should keep in mind that the present TRN data might still contain false positive edges as well as be missing false negative ones. Nevertheless, this comparison suggests that TF gene expression profile information and computational prediction of TFBSs are not sufficient to unravel TRN architecture. Instead, the present Matrix RNAi approach could be of great help to depict a high quality tissue- or cell type-specific TRN. Although this approach has been used here to depict a TRN in a stable state as a snapshot, the Matrix RNAi analysis, if it were performed at multiple time points, should also be a very powerful tool to uncover even dynamically changing TRNs during differentiation and development as a flipbook composed of a succession of TRN snapshots.

## Materials and methods

### siRNA

Two stealth siRNAs (Invitrogen, Carlsbad, CA, USA) were tested for each target gene and the one that gave the higher level of knockdown was used for the Matrix RNAi. siRNAs selected are listed in Additional data file 1. Stealth RNAi Negative universal control MED (Invitrogen) was used as the calibrator siRNA.

### Cell culture, siRNA transfection and RNA extraction

THP-1 cells were cultured in RPMI1640 (Invitrogen), 10% fetal bovine serum, penicillin/streptomycin (Invitrogen), 10 mM HEPES, 1 mM sodium pyruvate and 50 μM 2-mercaptoethanol. Reverse transfection of 1 × 10^6 ^cells in each 60-mm cell culture dish was performed with 20 nM (final concentration) of each stealth siRNA, Opti-MEM (Invitrogen) and 1.6 μg/ml (final concentration) of Lipofectamine 2000 (Invitrogen) according to the manufacturer's instructions. RNAs were extracted 48 h after transfection with FastPure RNA kit (TAKARA BIO, Kyoto, Japan) according to the manufacturer's instructions. RNA was quantified with NanoDrop (NanoDrop Technologies, Wilmington, DE, USA). All siRNA transfection experiments were performed in biological quadruplicate and the siRNA-treated cells in two dishes were combined as one lot to extract RNA samples.

### Expression analysis

Expression levels of TF genes in the cells treated with the specific siRNAs or the calibrator negative control siRNA were estimated by qRT-PCR in triplicate with the specific primer sets (Additional data file 1). Glyceraldehyde-3-phosphate dehydrogenase (GAPDH) mRNA level was determined with a specific primer set as an internal expression control. A total of four biological replicates were assayed. Reverse transcription reaction was performed with PrimeScript RT-PCR Kit (Perfect Real Time, TAKARA BIO) and GeneAmp PCR System 9700 (Applied Biosystems, Foster City, CA, USA) according to the manufacturer's instructions. qRT-PCR was done in 10-μl reaction mixture with SYBR Premix Ex Taq™ (Perfect Real Time, TAKARA BIO) on an ABI 7500 Fast real time PCR system (Applied Biosystems). PCR parameters consisted of heating at 94°C for 5 s, followed by 40 cycles of 94°C for 5 s and 62.5°C for 20 s. The relative amount (expression ratio) of the target gene mRNA was normalized to the endogenous GAPDH mRNA using the 2^-ΔΔCT ^method [37]. The difference in the threshold cycle of a sample (ΔC_T _(sample)) was calculated by subtracting the threshold cycle of GAPDH mRNA from that of the target TF mRNA in the RNA samples extracted from THP-1 cells transfected with the target TF-specific siRNA. The difference in the threshold cycle of the calibrator (ΔC_T _(calibrator)) was calculated by subtracting the threshold cycle of GAPDH mRNA from that of the target TF mRNA in the RNA samples extracted from THP-1 cells transfected with negative control siRNA. ΔΔC_T _was calculated by subtracting ΔC_T _(sample) from ΔC_T _(calibrator) and 2^-ΔΔC^_T _indicates the expression ratio. Standard deviation (SD) of ΔΔC_T _in a total of four biological replicates was calculated and the cutoff value was arbitrarily defined as 2 SD above the mean. To evaluate the interferon response that might be induced by siRNA administration, we examined the levels of expression of several interferon-responsive genes, such as *OAS1*. However, no significant changes in their expression levels were detected.

### Transcription factor binding assay by X-ChIP/qRT-PCR

The procedures for X-ChIP were essentially as described previously [5,7,14] with minor modifications. The soluble chromatin prepared from 1 × 10^7 ^cells was incubated with specific antibody against each TF (Additional data file 11) for more than 12 h at 4°C. The chromatin-antibody mixture was incubated with Dynabeads Protein G (Dynal Biotech, Oslo, Norway) for 1 h at 4°C and the immunoprecipitates were captured by using magnets. The immunoprecipitates recovered were washed once with IP wash buffer Low Salt (2 mM EDTA, 20 mM Tris-HCl pH 8.0, 150 mM NaCl, 1% Triton X-100, 0.1% SDS), once with IP wash buffer High Salt (2 mM EDTA, 20 mM Tris-HCl pH 8.0, 500 mM NaCl, 1% Triton X-100, 0.1% SDS), once with IP wash buffer LiCl (1 mM EDTA, 10 mM Tris-HCl pH 8.0, 250 mM LiCl, 0.5% NP-40, 0.5% sodium deoxycholate) and twice with TE buffer (10 mM Tris-HCl pH 8.0, 1 mM EDTA). The washed protein-DNA complexes were released from Dynabeads Protein G twice with 250 μl of elution buffer (100 mM sodium bicarbonate, 1% SDS). NaCl was added to the protein-DNA complexes in a final concentration of 20 mM, then the mixture was incubated at 65°C for 3.5 h for reversal of formaldehyde-induced cross-linking and treated with 0.05 mg/ml RNase A (Nippon Gene, Tokyo, Japan) at 65°C for 30 minutes. After the addition of Tris-HCl (pH 6.8) and EDTA (pH 8.0) to final concentrations of 40 and 10 mM, respectively, the reversed samples were treated with 0.25 mg/ml protease K (Nippon Gene) at 45°C for 1 h. The DNA released was then extracted with phenol and phenol:chloroform:isoamylalcohol (25:24:1), isopropanol-precipitated with Ethachinmate (Nippon Gene), and then dissolved in 250 μl of H_2_O. DNA samples obtained by ChIP with each specific antibody and from the precipitates obtained without any antibody and input DNA (total chromatin DNA) were used as templates for qRT-PCR assay. The procedures of qRT-PCR were essentially the same as those described in the section on qRT-PCR.

### Expression analysis

Enrichment of the target DNA fragments was assessed with ΔC_T _obtained by subtraction of the C_T_values observed for the ChIP samples with specific antibody from the C_T_s observed without any antibody. Sequences of the primers used for ChIP/qRT-PCR were described in Additional data file 12. ChIP experiments were performed in triplicate with each different batch of chromatin preparations and qRT-PCRs were performed in triplicate with each primer set. The ΔC_T _values obtained were averaged for each of the TF-TF gene edge pairs. For evaluation of DNA fragment enrichment in the experiments, we set the threshold to 1.0 ΔC_T _for all the triplicate experiments.

### Statistics

To evaluate the significance of RNAi knockdowns, perturbations and enrichment of the specific DNA fragments that were bound by each of the TF proteins, a two-tailed Student's *t*-test was used for generating *P*-values. In all analyses the threshold for statistical significance was *P *< 0.05. Because multiple testing errors were expected, FDR analysis was performed with the *P*-values computed from the Student *t*-tests by applying the Storey correction approach [38] with the QVALUE program [39] and the R software environment for statistical computing [40]. In practice, a cutoff for null hypothesis rejection was set to 0.05 to ensure a 5% FDR.

## Abbreviations

AML: acute myeloid leukemia; ChIP: chromatin immunoprecipitation; FDR: false discovery rate; GAPDH: glyceraldehyde-3-phosphate dehydrogenase; PMA: phorbol 12-myristate 13-acetate; qRT-PCR: quantitative real-time RT-PCR; RNAi: RNA interference; SD: standard deviation; siRNA: small interfering RNA; TF: transcription factor; TFBS: transcription factor binding site; TRN: transcriptional regulatory network; TSS: transcription start site.

## Authors' contributions

YT designed the research, and performed whole knockdown experiments, expression analysis, ChIP and overall analysis. CS carried out whole knockdown experiments, expression analysis and ChIP. HM carried out ChIP-qRT-PCR and ChIP analysis. ARRF carried out computational analysis of TFBS prediction. AK carried out the western blot confirmation of knockdown. MS and YH were involved in the conceptualization of this project. YT and ARRF wrote the manuscript. MS edited the manuscript.

## Additional data files

The following additional data are available with the online version of this paper: a table listing primer and siRNA sequences, TF knockdown and expression changing patterns during PMA-induced THP-1 cellular differentiation (Additional data file 1); a figure showing assessment of knockdown efficiency at the protein level (Additional data file 2); figures showing overviews of significant and non-significant edges (Additional data file 3); a table listing qRT-PCR data used for the Matrix RNAi analysis before selection (Additional data file 4); a table of all selected significant qRT-PCR data used for Matrix RNAi analysis (Additional data file 5); tables listing significant perturbation edges detected by Matrix RNAi analysis (Additional data file 6); figures showing perturbation networks from all selected significant edges (Additional data file 7); figures showing comparisons of double knockdown and single knockdown of MYB and MLLT3 (Additional data file 8); a table listing X/ChIP-qRT-PCR results (Additional data file 9); Venn diagrams for comparison between several criteria to extract potential regulatory edges (Additional data file 10); a table listing antibodies for X/ChIP-qRT-PCR (Additional data file 11); a table listing primer sequences and positions for X/ChIP-qRT-PCR (Additional data file 12).

## Supplementary Material

Additional data file 1 This table shows siRNA and primer sequences for the knockdown analysis, expression data (ΔC_T _value) and knockdown efficiencies of siRNAs targeting a TF gene in the Matrix RNAi analysis. Expression patterns analyzed in FANTOM4 were classified into pro-differentiated, anti-differentiated, static, transient and dynamic according to the expression profiles of the TF genes examined in the present Matrix RNAi research: 'static', 'transient' and 'dynamic' represent the profiles in which the expression level of a given TF gene is constant or significantly unchanged, having one peak or valley, and having multiple peaks and/or valleys, respectively, during PMA-induced THP-1 differentiation. Click here for file

Additional data file 2 Western blotting with specific antibodies against each of the TFs and the protein extracts prepared from the THP-1 cells transfected with 20 nM negative control siRNA or each of the TF-specific siRNAs was carried out to evaluate TF knockdown efficiency at the protein level. Control: protein extracts from THP-1 cells transfected with negative control siRNA. siRNA: protein extracts from THP-1 cells transfected with each TF-specific siRNA. The levels of actin and TATA binding protein (TBP) were also examined as internal references (controls 1 and 2, respectively). Click here for file

Additional data file 3 Figure S1: distribution of perturbation magnitudes between significant and non-significant edges. The 927 edges and 83 auto-perturbation edges that corresponded to 78 TF and 5 non-TF genes that were knocked down and had a low SD (mean > 2 SD) and a low *P*-value (*P *< 0.05) in Student's *t*-test were selected as significant edge candidates. The remaining 6,958 edges were grouped together as non-significant edges. The edges in each group were divided according to their perturbation magnitudes, which were calculated on the basis of the data from the qRT-PCR assay (see qRT-PCR in Expression analysis, Materials and methods for details). Perturbation magnitude was represented by absolute ΔΔC_T_, in every 0.2 absolute ΔΔC_T _and the percentages of the number of edges in each fraction to the total number of the edges were plotted. Red bars represent the percentage of the number of significant perturbation edges, black bars non-significant ones and yellow bars TF genes knocked down. Figure S2: magnitude of perturbation for significant and non-significant groups. Mean and SD values of ΔΔC_T_s of high (< 2 SD and *P *> 0.05) and low (> 2 SD and *P *< 0.05) SD and *P*-value groups were calculated. ΔΔC_T _values for knockdown of the TF genes (siRNA) are much larger than perturbation magnitudes, indicating that the influence of knockdown of TF genes on their downstream TF genes tends to be attenuated. Click here for file

Additional data file 4 All qRT-PCR data used for the Matrix RNAi analysis. ddCt indicates the average ddCt from four biological replicates. SD indicates the standard deviation of ddCt from four biological replicates. Ttest indicates the *P*-value of the dCt between the knockdown and control siRNA transfected samples. Click here for file

Additional data file 5 The 927 edges and 83 autoregulatory edges that showed a low SD (mean > 2 SD) and a low *P*-value (*P *< 0.05) in Student's-*t*-test were selected as significant edge candidates. The remaining 6,958 edges were removed from the qRT-PCR data. Click here for file

Additional data file 6 THP-1 cells (1 × 10^6 ^cells) were transfected with an individual siRNA species against each of the TF genes. The total RNA was extracted 48 h after the transfection and used for qRT-PCR. The changes in expression levels (perturbations) were evaluated by C_T _calculated according to the method described by Livak *et al*. [37]. Quadruplicated experiments were carried out to obtain the average C_T_, SD and *P*-value. Only the edges that gave a low SD (mean of ΔΔC_T _> 2 SD) and *P*-value (< 0.05) were selected as significant regulatory TF-TF gene edges for preparing this table. 'Input gene' and 'target gene' indicates genes knocked down by a specific siRNA and genes perturbed significantly after siRNA transfection, respectively. 'Activate' and 'suppress' indicate that knockdown of one TF led to a significant decrease in the expression of another and to a significant increase in the expression of another, respectively. The actual data for RNAi perturbation are in Additional data file 4 (for all of the edges tested) and Additional data file 5 (for only significant edges). Click here for file

Additional data file 7 For depiction of the putative networks, only significant edges (> 2 SD and *P *< 0.05) were extracted based on the Matrix RNAi data in Additional data files 4 and 5. The network was drawn by Cytoscape [33]. In these networks, TFs and TF genes regulated by them are not distinguished from each other, but the nodes emitting and accepting an arrow represent the putative regulators and regulated genes, respectively. Figure S1: perturbation network of significant regulatory edges based on Matrix RNAi data. Figure S2: pro-differentiative edge network. Figure S3: anti-differentiative edge network. Click here for file

Additional data file 8 Figure S1: comparison of the extent of THP-1 cell adhesion between individual knockdown of either MYB or MLLT3 and their double knockdown. Blue bars indicate floating cells and red bars indicate attached cells counted 96 h after siRNA transfection. M & M indicates double knockdown. NC indicates cells transfected with siRNA negative control. Figure S2: Venn diagram of genes affected by knockdown of MYB and MLLT3 and their double knockdown. Click here for file

Additional data file 9 Only the data showing a positive TF binding (ΔC_T _> 1.0 corresponding to two-fold enrichment of the TF-specific DNA fragments and *P *< 0.05 in Student's *t*-test) in three separate X-ChIP/qPCR experiments are indicated. Click here for file

Additional data file 10 Figure S1: comparison between *P*-value threshold (*P *< 0.05), q-value threshold (q-value < 0.05) and 2-SD threshold (a signal average < 2 × SD). Figure S2: comparison between 2-SD/*P*-value threshold and 2-SD/*P*-value threshold and ChIP/qPCR confirmation. Figure S3: comparison between 2-SD/*P*-value/q-value threshold and 2-SD/*P*-value/q-value threshold and ChIP/qPCR confirmation. FDR (q-value) was calculated by using the QVALUE program and R software as described in Materials and methods. The number in parentheses indicates the number of edges excluding auto-perturbation edges. Figure S4: accumulative number of TF-binding positive and negative edges with q-value. Regulatory edges tested for TF-binding were separated into two groups for significance in ChIP assay (ChIP-negative and -positive) and the numbers determined together with the q-values for their perturbations. Click here for file

Additional data file 11 Antibodies used in western blotting and X-ChIP analysis. Click here for file

Additional data file 12 Primers used in X-ChIP/qPCR analysis. Primer pairs were designed around 500 nt upstream from the TSSs of the respective genes, except for some genes that were difficult to design their primers in the very proximal region. Click here for file
