# Supplementary material for: Regulatory interdependence of myeloid transcription factors revealed by Matrix RNAi analysis
Source: Genome Biol. 2009 Nov 2;10(11):R121. doi: 10.1186/gb-2009-10-11-r121 (PMC2810662; doi:10.1186/gb-2009-10-11-r121)
Supplement: Additional data file 2 — Western blotting with specific antibodies against each of the TFs and the protein extracts prepared from the THP-1 cells transfected with 20 nM negative control siRNA or each of the TF-specific siRNAs was carried out to evaluate TF knockdown efficiency at the protein level. Control: protein extracts from THP-1 cells transfected with negative control siRNA. siRNA: protein extracts from THP-1 cells transfected with each TF-specific siRNA. The levels of actin and TATA binding protein (TBP) were also examined as internal references (controls 1 and 2, respectively). [file gb-2009-10-11-r121-S2.PPT]

## Slide 1
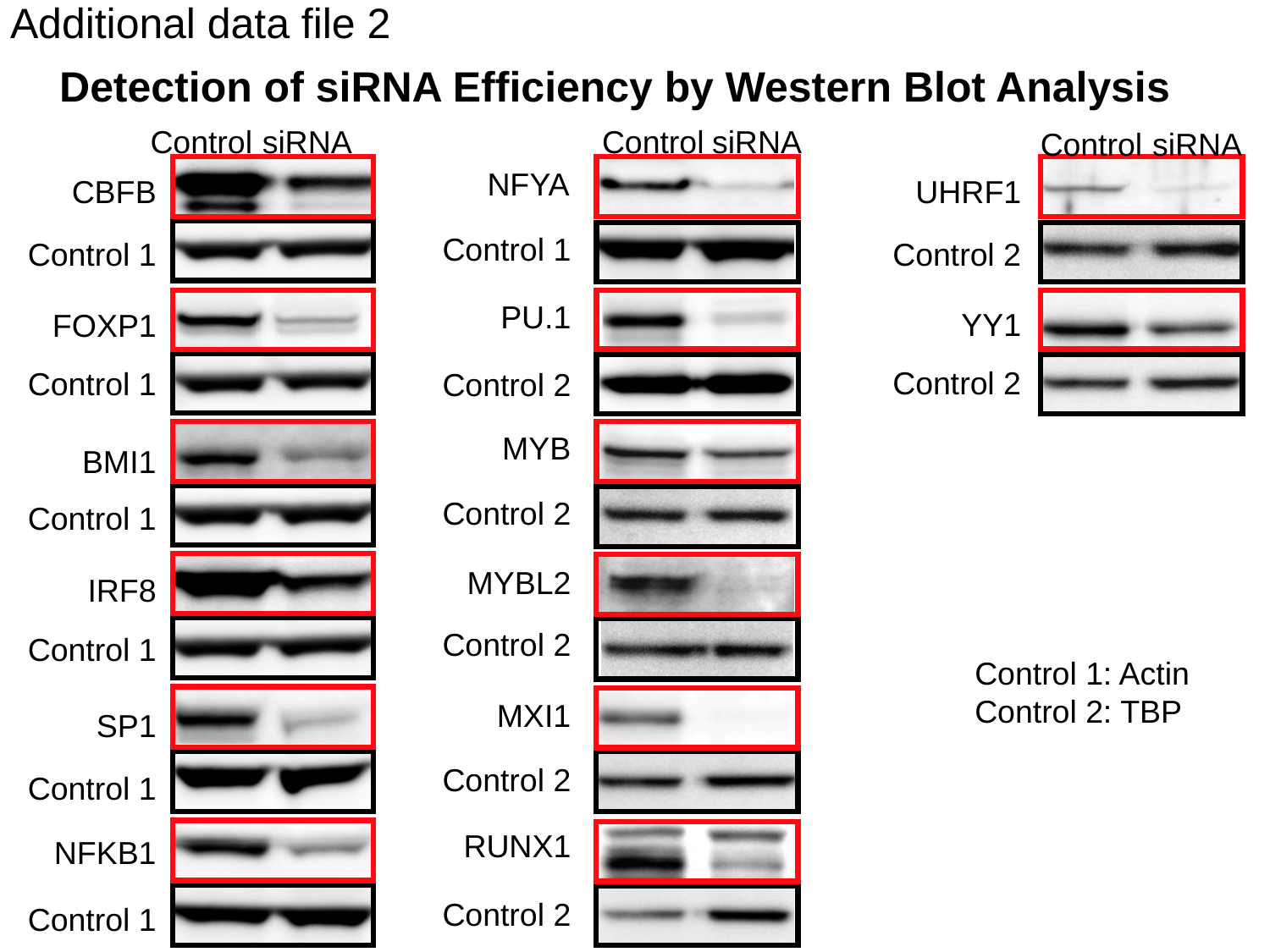

Additional data file 2
Detection of siRNA Efficiency by Western Blot Analysis
Control
siRNA
Control
siRNA
Control
siRNA
NFYA
CBFB
UHRF1
Control 1
Control 1
Control 2
PU.1
YY1
FOXP1
Control 2
Control 1
Control 2
MYB
BMI1
Control 2
Control 1
MYBL2
IRF8
Control 2
Control 1
Control 1: Actin
Control 2: TBP
MXI1
SP1
Control 2
Control 1
RUNX1
NFKB1
Control 2
Control 1
