# Supplementary material for: Regulatory interdependence of myeloid transcription factors revealed by Matrix RNAi analysis
Source: Genome Biol. 2009 Nov 2;10(11):R121. doi: 10.1186/gb-2009-10-11-r121 (PMC2810662; doi:10.1186/gb-2009-10-11-r121)
Supplement: Additional data file 3 — Figure S1: distribution of perturbation magnitudes between significant and non-significant edges. The 927 edges and 83 auto-perturbation edges that corresponded to 78 TF and 5 non-TF genes that were knocked down and had a low SD (mean > 2 SD) and a low P-value (P < 0.05) in Student's t-test were selected as significant edge candidates. The remaining 6,958 edges were grouped together as non-significant edges. The edges in each group were divided according to their perturbation magnitudes, which were calculated on the basis of the data from the qRT-PCR assay (see qRT-PCR in Expression analysis, Materials and methods for details). Perturbation magnitude was represented by absolute ΔΔCT, in every 0.2 absolute ΔΔCT and the percentages of the number of edges in each fraction to the total number of the edges were plotted. Red bars represent the percentage of the number of significant perturbation edges, black bars non-significant ones and yellow bars TF genes knocked down. Figure S2: magnitude of perturbation for significant and non-significant groups. Mean and SD values of ΔΔCTs of high (< 2 SD and P > 0.05) and low (> 2 SD and P < 0.05) SD and P-value groups were calculated. ΔΔCT values for knockdown of the TF genes (siRNA) are much larger than perturbation magnitudes, indicating that the influence of knockdown of TF genes on their downstream TF genes tends to be attenuated. [file gb-2009-10-11-r121-S3.PPT]

## Slide 1
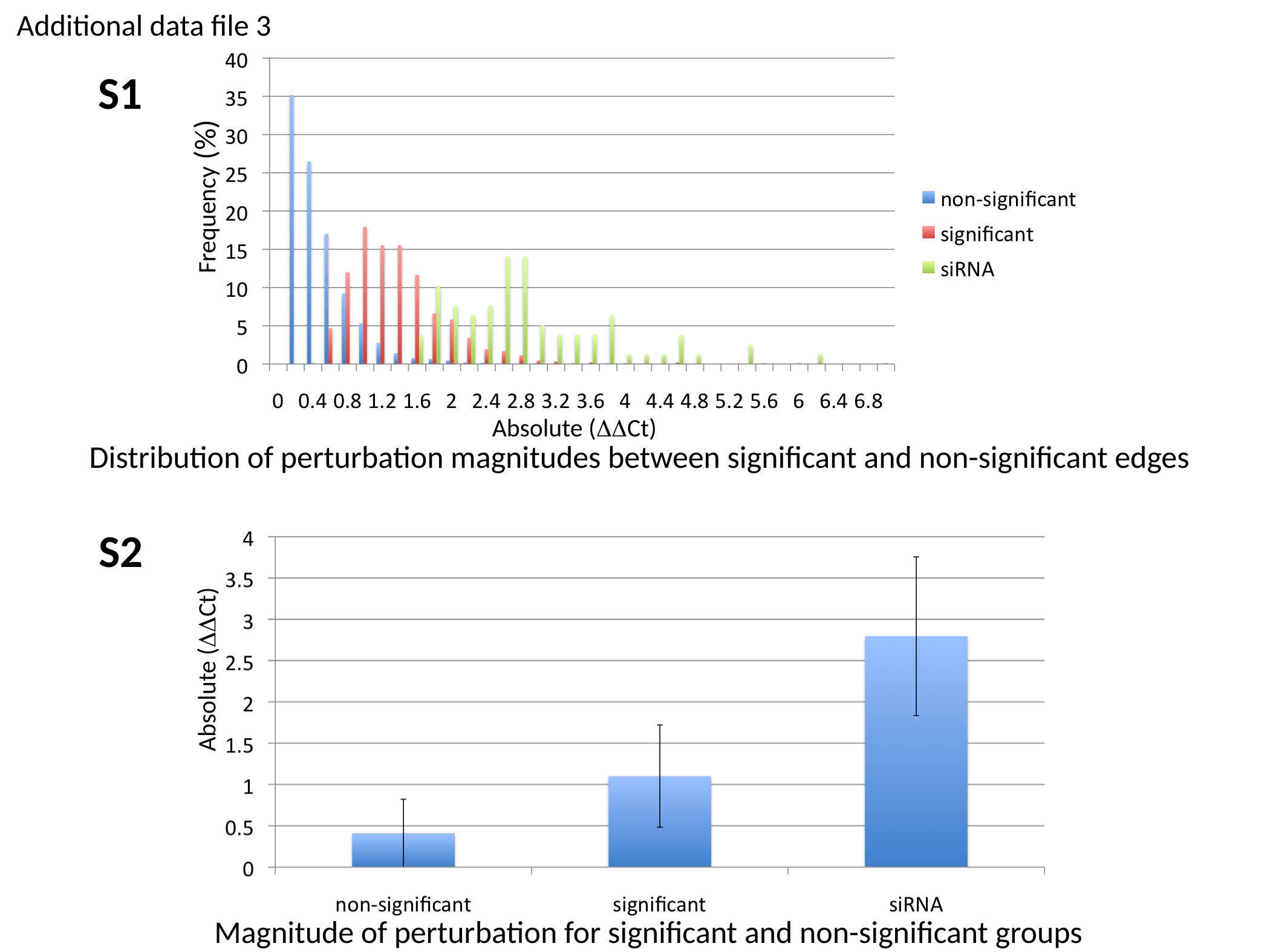

Additional data file 3
S1
Frequency (%)
Absolute (ΔΔCt)
Distribution of perturbation magnitudes between significant and non-significant edges
S2
Absolute (ΔΔCt)
Magnitude of perturbation for significant and non-significant groups
