# Supplementary material for: Regulatory interdependence of myeloid transcription factors revealed by Matrix RNAi analysis
Source: Genome Biol. 2009 Nov 2;10(11):R121. doi: 10.1186/gb-2009-10-11-r121 (PMC2810662; doi:10.1186/gb-2009-10-11-r121)
Supplement: Additional data file 7 — For depiction of the putative networks, only significant edges (> 2 SD and P < 0.05) were extracted based on the Matrix RNAi data in Additional data files 4 and 5. The network was drawn by Cytoscape [33]. In these networks, TFs and TF genes regulated by them are not distinguished from each other, but the nodes emitting and accepting an arrow represent the putative regulators and regulated genes, respectively. Figure S1: perturbation network of significant regulatory edges based on Matrix RNAi data. Figure S2: pro-differentiative edge network. Figure S3: anti-differentiative edge network. [file gb-2009-10-11-r121-S7.PPT]

## Slide 1
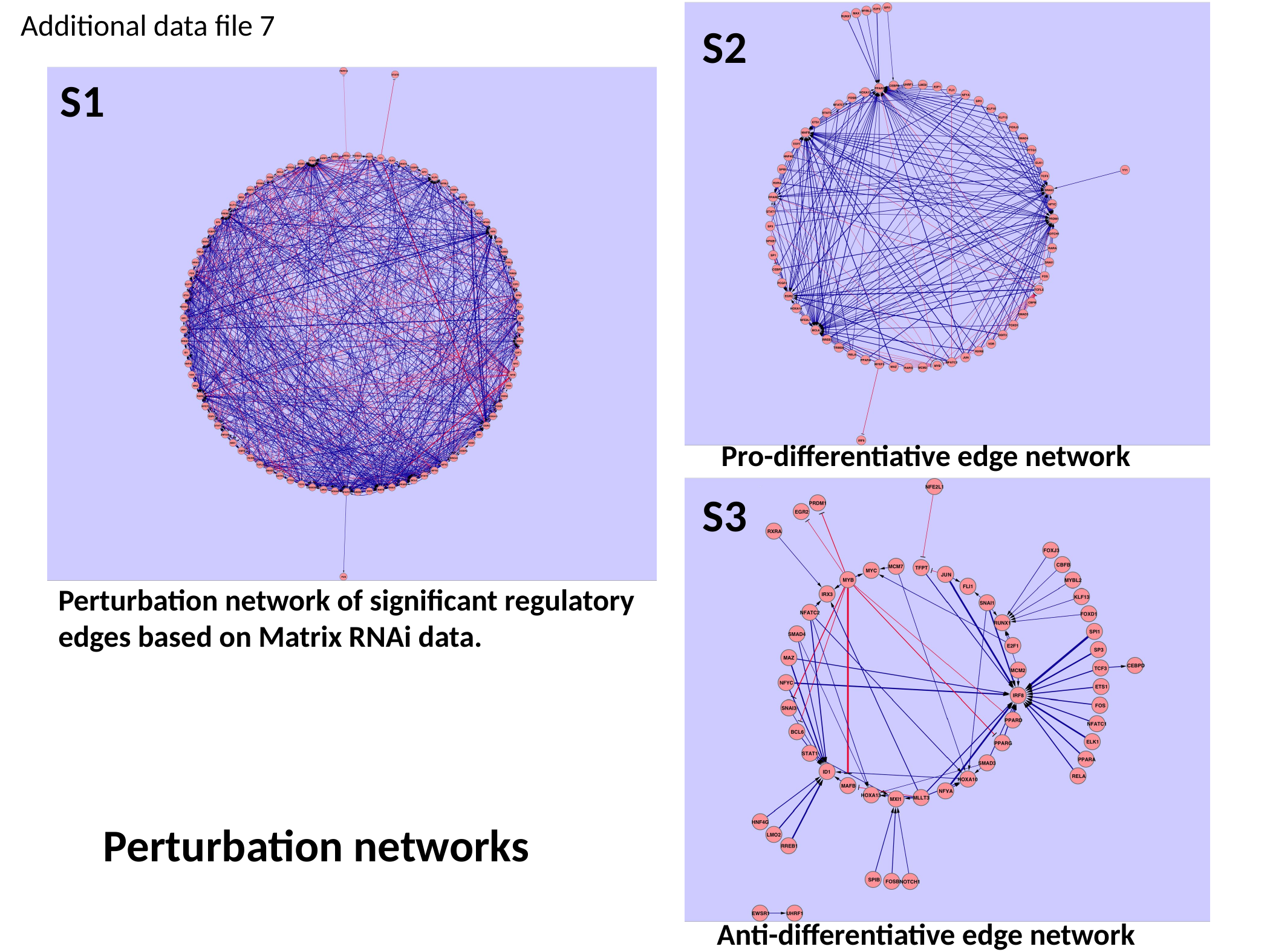

Additional data file 7
S2
S1
Pro-differentiative edge network
S3
Perturbation network of significant regulatory edges based on Matrix RNAi data.
Perturbation networks
Anti-differentiative edge network
