# Supplementary material for: Regulatory interdependence of myeloid transcription factors revealed by Matrix RNAi analysis
Source: Genome Biol. 2009 Nov 2;10(11):R121. doi: 10.1186/gb-2009-10-11-r121 (PMC2810662; doi:10.1186/gb-2009-10-11-r121)
Supplement: Additional data file 8 — Figure S1: comparison of the extent of THP-1 cell adhesion between individual knockdown of either MYB or MLLT3 and their double knockdown. Blue bars indicate floating cells and red bars indicate attached cells counted 96 h after siRNA transfection. M & M indicates double knockdown. NC indicates cells transfected with siRNA negative control. Figure S2: Venn diagram of genes affected by knockdown of MYB and MLLT3 and their double knockdown. [file gb-2009-10-11-r121-S8.PPT]

## Slide 1
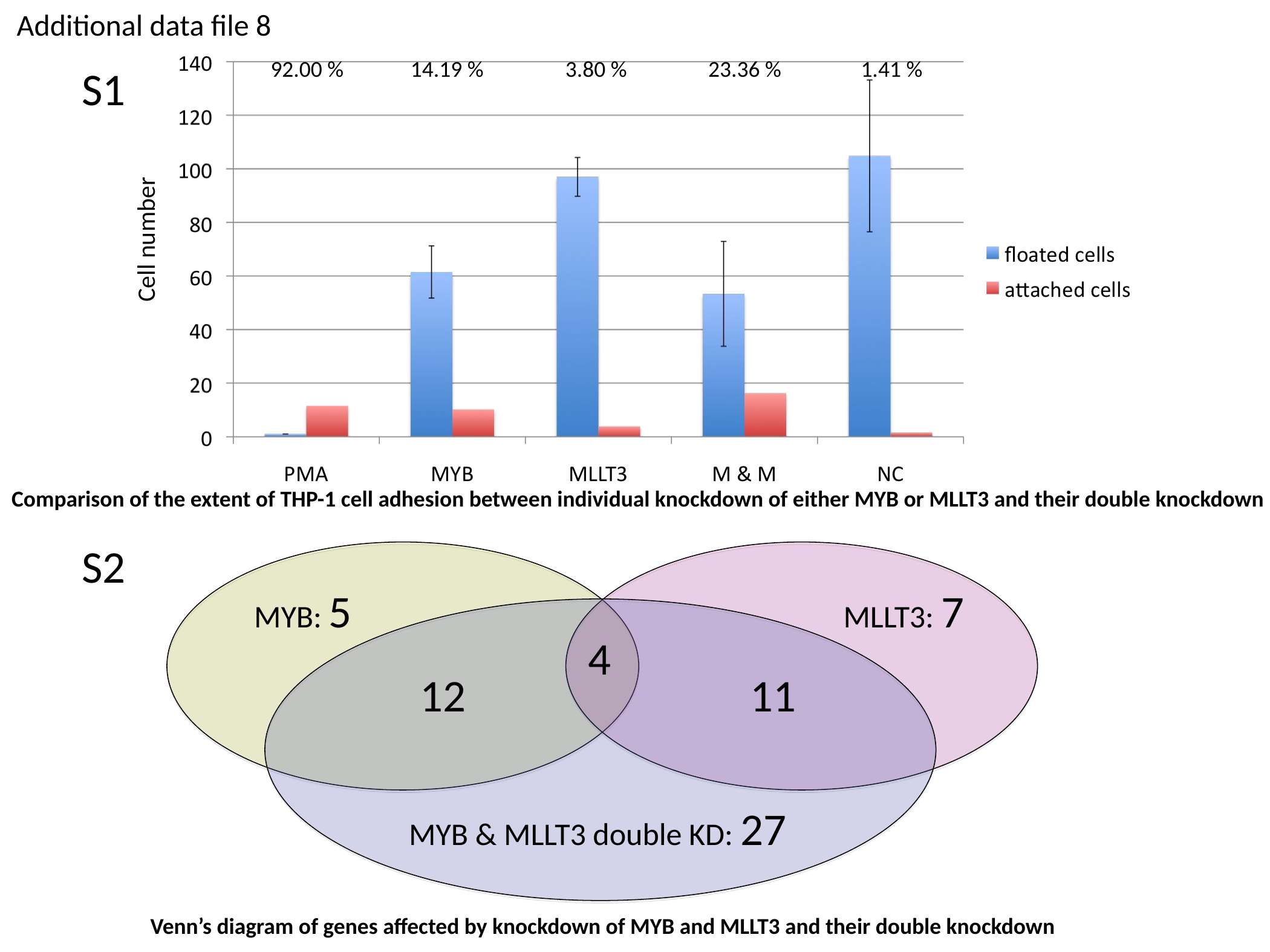

Additional data file 8
92.00 %
14.19 %
3.80 %
23.36 %
1.41 %
S1
Cell number
Comparison of the extent of THP-1 cell adhesion between individual knockdown of either MYB or MLLT3 and their double knockdown
S2
MYB: 5
MLLT3: 7
4
12
11
MYB & MLLT3 double KD: 27
Venn’s diagram of genes affected by knockdown of MYB and MLLT3 and their double knockdown
