# Supplementary material for: Regulatory interdependence of myeloid transcription factors revealed by Matrix RNAi analysis
Source: Genome Biol. 2009 Nov 2;10(11):R121. doi: 10.1186/gb-2009-10-11-r121 (PMC2810662; doi:10.1186/gb-2009-10-11-r121)
Supplement: Additional data file 10 — Figure S1: comparison between P-value threshold (P < 0.05), q-value threshold (q-value < 0.05) and 2-SD threshold (a signal average < 2 × SD). Figure S2: comparison between 2-SD/P-value threshold and 2-SD/P-value threshold and ChIP/qPCR confirmation. Figure S3: comparison between 2-SD/P-value/q-value threshold and 2-SD/P-value/q-value threshold and ChIP/qPCR confirmation. FDR (q-value) was calculated by using the QVALUE program and R software as described in Materials and methods. The number in parentheses indicates the number of edges excluding auto-perturbation edges. Figure S4: accumulative number of TF-binding positive and negative edges with q-value. Regulatory edges tested for TF-binding were separated into two groups for significance in ChIP assay (ChIP-negative and -positive) and the numbers determined together with the q-values for their perturbations. [file gb-2009-10-11-r121-S10.PPT]

## Slide 1
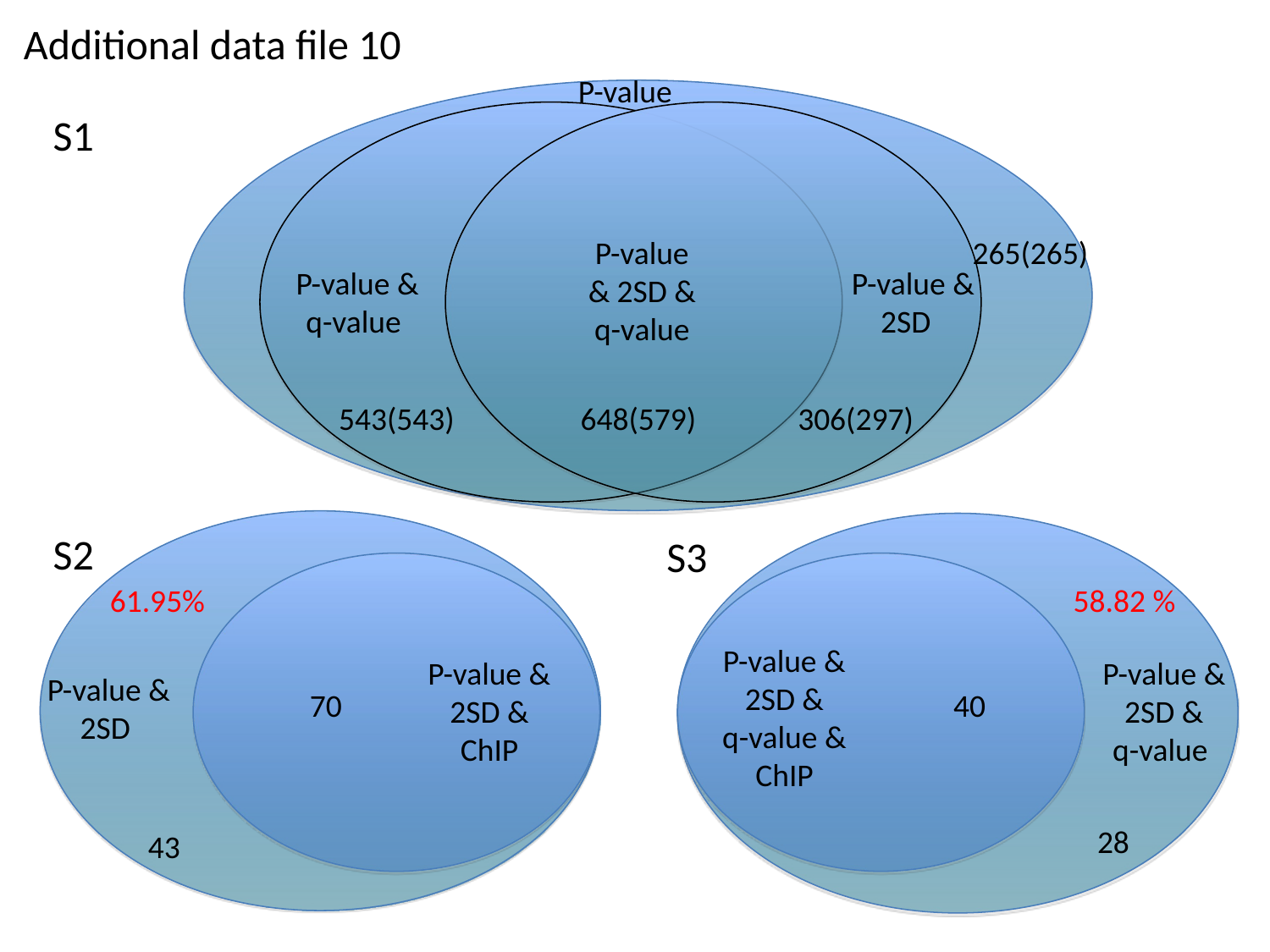

Additional data file 10
P-value
S1
P-value & 2SD &
q-value
265(265)
P-value &
q-value
P-value & 2SD
543(543)
648(579)
306(297)
S2
S3
61.95%
58.82 %
P-value & 2SD &
q-value &
ChIP
P-value & 2SD &
ChIP
P-value &
2SD &
q-value
P-value & 2SD
70
40
28
43

## Slide 2
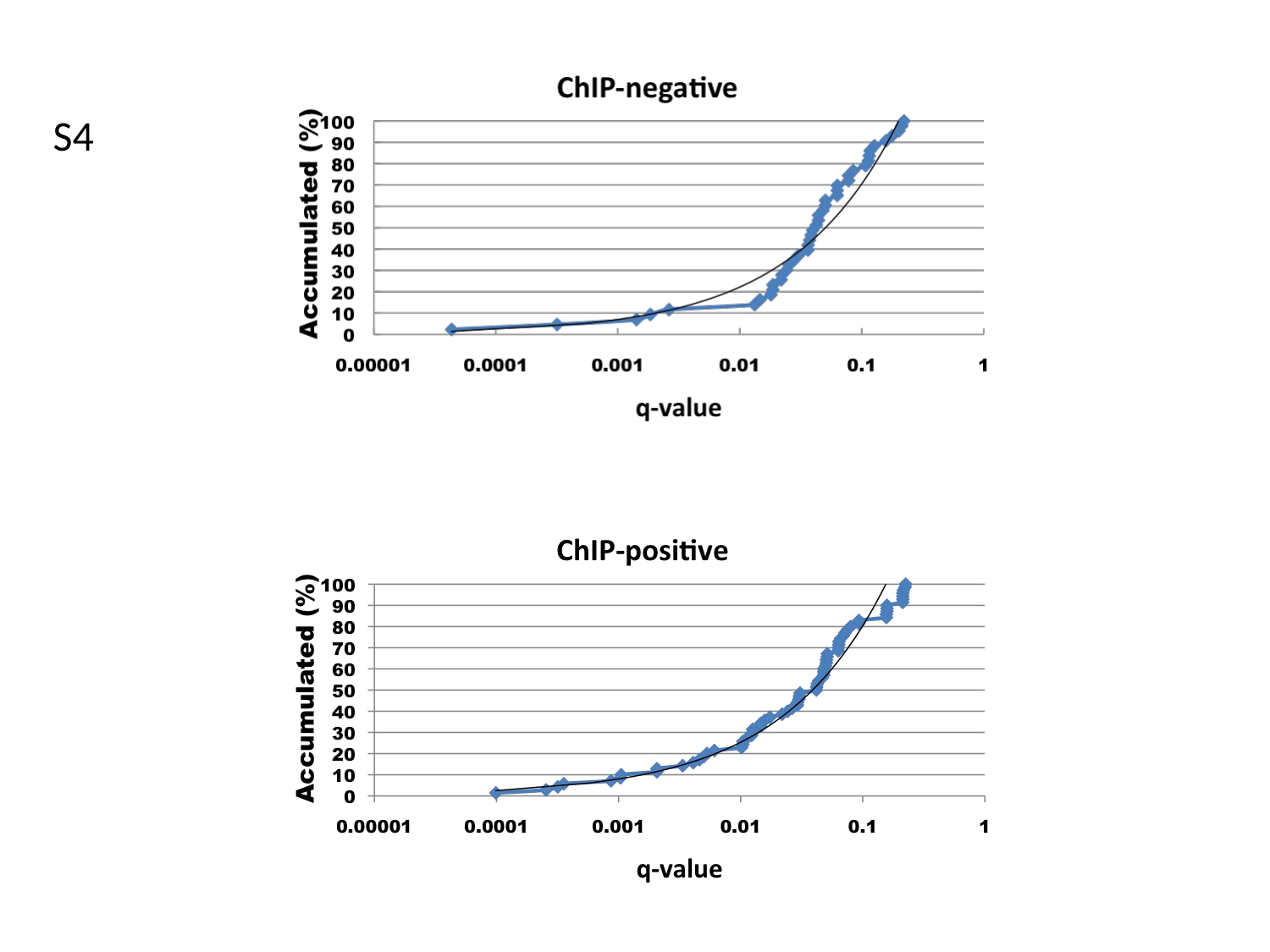

S4
